# Impact of Mean Blood Pressure Profiles in Percutaneous Left Ventricular Assist Device‐Supported High‐Risk Percutaneous Coronary Intervention: The PROTECT III Study

**DOI:** 10.1161/JAHA.124.036367

**Published:** 2025-05-15

**Authors:** Guillaume Bonnet, Karl‐Philipp Rommel, Batla Falah, Alexandra J. Lansky, Yiran Zhang, Michael J. Schonning, Björn Redfors, Daniel Burkhoff, David J. Cohen, M. Babar Basir, William W. O'Neill, Juan F. Granada

**Affiliations:** ^1^ Cardiovacular Research Foundation New York NY USA; ^2^ Medico‐Surgical Department Hopital Cardiologique Haut‐Lévêque, Bordeaux University Hospital Bordeaux France; ^3^ Department of Cardiology Heart Center at University of Leipzig and Leipzig Heart Institute Leipzig Germany; ^4^ Department of Cardiology Yale School of Medicine New Haven CT USA; ^5^ Barts Heart Centre London and Queen Mary University of London London United Kingdom; ^6^ Department of Population Health Sciences Weill Cornell Medicine New York NY USA; ^7^ Department of Molecular and Clinical Medicine Gothenburg University Gothenburg Sweden; ^8^ Department of Cardiology Sahlgrenska University Hospital Gothenburg Sweden; ^9^ St. Francis Hospital Roslyn NY USA; ^10^ Center for Structural Heart Disease, Division of Cardiology Henry Ford Health System Detroit MI USA

**Keywords:** blood pressure control, high‐risk percutaneous coronary intervention, mean arterial pressure, PROTECT III, temporary left ventricular assist device support, Percutaneous Coronary Intervention

## Abstract

**Background:**

Percutaneous left ventricular assist devices are used prophylactically to prevent hypotension during high‐risk percutaneous coronary intervention. However, the impact of preprocedural hemodynamic profiles on procedural and clinical outcomes in these patients is unknown.

**Methods and Results:**

Patients from the central venous access device PROTECT III registry (NCT04136392) were categorized according to preprocedural mean blood pressure (MBP). Procedural and in‐hospital outcomes, including hypotensive episodes, need for prolonged percutaneous left ventricular assist device support, and in‐hospital death, were compared between groups. We also assessed the relationship between preprocedural MBP and 90‐day major adverse cardiovascular and cerebrovascular events, which included all‐cause death, myocardial infarction, stroke/transient ischemic attack, and repeat revascularization, as well as with 1‐year mortality. A total of 1159 patients underwent percutaneous left ventricular assist device‐supported high‐risk percutaneous coronary intervention and were stratified into 4 hemodynamic profiles of preprocedural MBP level: MBP>100 mm Hg (n=242), >90 to ≤100 mm Hg (n=264), >80 to ≤90 mm Hg (n=306), and ≤80 mm Hg (n=347). Lower preprocedural MBP was associated with baseline anemia, history of heart failure, left main disease, and transfer from another hospital. In‐hospital and procedural adverse outcomes did not differ between the BP categories. However, 90‐day major adverse cardiovascular and cerebrovascular events rates and 1‐year mortality increased with decreasing baseline BP levels. The association between BP category and 1‐year mortality remained significant after adjustment for other factors (hazard ratio [HR], 0.79 [95% CI, 0.71–0.88], *P*<0.001).

**Conclusions:**

In a real‐world cohort undergoing high‐risk percutaneous coronary intervention with percutaneous left ventricular assist device support, there was no association between hemodynamic status and in‐hospital outcomes. Lower preprocedural BP was associated with higher rates of 90‐day major adverse cardiovascular and cerebrovascular events and 1‐year mortality.

**Registration:**

URL: https://www.clinicaltrials.gov; Unique identifier: NCT04136392.

**Nonstandard Abbreviations and Acronyms**
HRPCI: high‐risk percutaneous intervention
MACCE: major adverse cardiovascular and cerebrovascular events
MBP: mean blood pressure
MCS: mechanical circulatory support
pLVAD: percutaneous left ventricular assist device

**Clinical Perspective**

**What Is New?**

- Lower preprocedural mean blood pressure (MBP) was correlated with baseline anemia, heart failure history, and more severe clinical conditions in patients from the PROTECT III study.
- High intraprocedural hemodynamic stability is maintained in percutaneous left ventricular assist device‐supported high‐risk percutaneous coronary intervention, independent of preprocedural MBP, in patients from the PROTECT III study.
- Lower preprocedural MBP in percutaneous left ventricular assist device‐supported high‐risk percutaneous coronary interventions associated with higher 90‐day MACCE and 1‐year mortality rates in patients from the PROTECT III study.

**What Are the Clinical Implications?**

- There were no significant in‐hospital outcome differences across MBP groups in the PROTECT III study; however, lower preprocedural MBP correlated with higher 90‐day MACCE rates and 1‐year mortality, suggesting preprocedural hemodynamic status as a predictor of longer‐term post‐high‐risk percutaneous coronary intervention risks.

Advances in interventional cardiology have revolutionized the management of patients with complex coronary artery disease and severe left ventricular dysfunction, expanding the ability to apply for percutaneous coronary interventions to higher‐risk cohorts. These procedures are then termed "high‐risk percutaneous coronary interventions" (HRPCI).^1^ Although there is no standard definition, HRPCI is defined by overlapping and potentially additive features including patient comorbidities, hemodynamics compromise, depressed left ventricular ejection fraction (LVEF), and anatomic complexity.^2^ Given the diverse nature of these factors, this specific patient population is heterogeneous and remains insufficiently investigated. The heterogeneity of these patients, coupled with the variety of these factors, may lead to inadequate patient selection and the hampering of individualized care before, during, and after intervention.^3^, ^4^

One of the technological advancements that has expanded the ability to perform HRPCI is the advent of temporary percutaneous left ventricular assist devices (pLVADs), such as the Impella system (Abiomed, Danvers, MA, USA).^5^, ^6^ Percutaneous left ventricular assist device‐assisted PCI is designed to prevent hypotension and improve hemodynamic stability during these high‐risk procedures.^7^, ^8^ Although monitoring hemodynamics during the PCI procedure, particularly blood pressure (BP), is crucial in managing these devices, little is known about the relationship between preprocedural BP and short or longer‐term outcomes. In the context of all‐comers PCI, the preprocedural mean BP (MBP) has been demonstrated to predict long‐term outcomes.^9^ However, in the context of HRPCI with pLVAD support, the role of hemodynamics status and preprocedural MBP remains unknown.

Therefore, we sought to investigate the association of comprehensive hemodynamic profiles, including preprocedural MBP, with procedural, in‐hospital and longer‐term outcomes in patients undergoing pLVAD‐supported HRPCI in a multicenter, modern registry.

## METHODS

### Study Design and Patient Population

The PROTECT III study design, rationale, and selected results have been published previously.^10^, ^11^ Briefly, the PROTECT III study was an observational study of patients who underwent pLVAD‐supported HRPCI conducted in 46 centers in North America. It is a part of the global central venous access device registry (NCT04136392), which consists of several Food and Drug Administration‐audited post‐approval studies evaluating the safety and effectiveness of pLVAD for various indications.^12^ The PROTECT III study specifically examined patients without cardiogenic shock undergoing elective or urgent HRPCI procedures, where Impella 2.5 or Impella CP devices were implanted to maintain hemodynamic stability during the procedure. The study followed the standard of care set by the treating physician, including the definition of HRPCI, the decision to use Impella, the type of Impella device chosen, and the duration of mechanical circulatory support. Patients requiring pLVAD implantation as a rescue measure were not included in this study.

The study adhered to the principles outlined in the Declaration of Helsinki and protocol was approved from the institutional review board or independent ethics committee at each participating center before patient enrollment. A 12‐member steering committee oversaw the study, comprising interventional cardiologists, cardiac surgeons, and heart failure specialists. The sponsor (Abiomed Inc., Danvers, MA, USA) oversaw study data management and source document verification and the Cardiovascular Research Foundation (New York, NY, USA) received funding for statistical analysis. The authors had unrestricted access to the study data and assume responsibility for the accuracy and reliability of this report. Artificial intelligence was not used in any step of the data curation, data analysis, or in writing of the article. The data from this study may be available to support additional studies; such requests should be made to the corresponding author.

In‐hospital data were collected from admission to discharge, encompassing baseline characteristics, preprocedural echocardiography, details of percutaneous coronary intervention, and pLVAD‐related information. Subsequently, patients were followed up for a period of 90 days to assess additional outcomes, and all‐cause mortality was evaluated at 1 year. Baseline laboratory values were preprocedural and measured closest to the time of pLVAD placement during the hospital admission. The analysis of angiographic data, including the calculation of quantitative Synergy Between Percutaneous Coronary Intervention with Taxus and Cardiac Surgery (SYNTAX) and ischemia jeopardy scores, was performed by the angiographic core laboratory (Beth Israel Deaconess Medical Center, Boston, MA). All major adverse cardiac and cerebrovascular events (MACCE: including all‐cause death, myocardial infarction, stroke/transient ischemic attack, and repeat revascularization) were adjudicated by an independent clinical events committee.

### Blood Pressure Assessment

Patients underwent systematic evaluation of their hemodynamic status, including invasively measured BP levels at the beginning, during, and at the end of the procedure. This preprocedural pressure corresponded to the BP measured preimplantation of the Impella device. Patients were stratified according to clinically relevant cutoffs of preprocedural MBP: >100 mm Hg, >90 to ≤100 mm Hg, >80 to ≤90 mm Hg and ≤80 mm Hg.

### Study End Point Definitions

The primary study end point was all‐cause death at 1 year. The secondary end points were (1) 90‐day MACCE; (2) the individual components of 90‐day MACCE; (3) in‐hospital adverse events, including cardiogenic shock, ventricular arrhythmias, cardiac arrest, vascular/cardiac complications requiring surgery, limb ischemia, life‐threatening, disabling, or major bleeding (Bleeding Academic Research Consortium ≥3a), respiratory failure, and acute kidney injury; (4) the completeness of revascularization, as reflected by the change in SYNTAX score and ischemia jeopardy score from preprocedure to postprocedure; (5) immediate PCI‐related complications (defined as the composite of no reflow, abrupt closure, dissection, distal embolus, or coronary perforation); (6) the duration of post‐HRPCI hospitalization; and (7) severe hypotension. Detailed definitions of all study end points have been previously published.^11^

Severe hypotension was defined as systolic BP below 90 mm Hg for >5 minutes, requiring the administration of inotropic/vasopressor medications or intravenous fluids while the patient was supported with a hemodynamic support device.

### Statistical Analysis

Baseline characteristics are summarized as means±SD or medians with interquartile range for continuous measures and frequencies with proportions for categorical variables. Continuous variables were compared across the BP groups with the ANOVA, or the nonparametric Kruskal–Wallis test (in cases where the explanatory variables did not meet the assumptions of normality and variance homogeneity). Categorical variables were compared by the chi‐square test. For time‐to‐first event analyses, event rates were estimated by the Kaplan–Meier method and compared with log‐rank tests. The relationship between MBP and all‐cause mortality was represented by spline of Cox analysis hazard ratio (HR). Multivariable Cox proportional hazards models were constructed to adjust for potential confounders. The variables included in the multivariable Cox analysis for 1‐year mortality were selected based on prior evidence in the literature and their effect in univariable analysis. Multiple imputation using chained equations was used to handle covariates with missing data (Mice R package, using 20 sets of imputations); continuous parameters were imputed with random forest and categorical parameters with polynomial regressions. All *P* values are 2 tailed, and *P*<0.05 was considered statistically significant for all analyses. Statistical analyses were performed using SAS version 9.4 (SAS Institute Inc., Cary, NC, USA) and R version 2023.03.0+386 (R Foundation for Statistical Computing).

## RESULTS

### Baseline Patient Characteristics

Among 1237 patients undergoing pLVAD‐supported HRPCI in the PROTECT III registry from March 2017 to March 2020, 1159 patients (93.7%) had available preprocedural MBP data and were therefore included in the analysis population. There were no differences in baseline characteristics between patients with available preprocedural MBP data and those who were excluded due to missing data (Table S1, Figure S1).

At baseline, 347 (29.9%) patients had an MBP ≤80 mm Hg, 306 (26.4%) patients had MBP >80 to ≤90 mm Hg, 264 (22.8%) patients had an MBP of >90 to ≤100 mm Hg, and 242 (20.9%) patients had an MBP >100 mm Hg (Figure S2). Baseline characteristics by MBP group are reported in Table 1. There was no significant difference in age or sex between MBP groups. Body mass index increased progressively with increasing baseline MBP (from 27.9±6.4 kg/m^2^ in those with MBP ≤80 mm Hg to 29.3±6.7 kg/m^2^ in those with MBP >100 mm Hg, *P*<0.001). The distribution of patient comorbidities was similar between groups except for baseline anemia, which was more frequent in patients with the highest and lowest MBP. Additionally, a history of heart failure was more prevalent in patients with the lower MBP (66.2% of patients with MBP ≤80 mm Hg versus 61.4% of those with MBP >80 to ≤90 mm Hg versus 55.5% of those with MBP >90 to ≤100 mm Hg versus 56.3% of those with >100 mm Hg, *P*=0.03).

**Table 1:** Baseline Characteristics

	≤80 mm Hg (N=347)	>80 to ≤90 mm Hg (N=306)	>90 to ≤100 mm Hg (N=264)	>100 mm Hg (N=242)	*P* value	No.
Demographics						
Age, y	71.9±10.6 (n=347)	70.4±11.1 (n=306)	71.1±11.3 (n=264)	70.7±11.5 (n=242)	0.38	1159
Sex, male	72.0% (250/347)	72.9% (223/306)	75.8% (200/264)	74.0% (179/242)	0.76	1159
Race						1159
White	68.0% (236/347)	69.9% (214/306)	70.8% (187/264)	56.2% (136/242)	0.001	
Black or African American	11.5% (40/347)	12.4% (38/306)	9.9% (26/264)	18.6% (45/242)	0.02	
Asian	4.0% (14/347)	2.9% (9/306)	3.4% (9/264)	2.0% (5/242)	0.60	
American Indian/Alaska native	0.6% (2/347)	0.3% (1/306)	0.4% (1/264)	0.8% (2/242)	0.90	
Native Hawaiian/Other Pacific Islander	0.3% (1/347)	0% (0/306)	0% (0/264)	0% (0/242)	1.00	
Other race	3.8% (13/347)	3.6% (11/306)	2.7% (7/264)	4.1% (10/242)	0.82	
Unknown race	11.8% (41/347)	10.8% (33/306)	12.9% (34/264)	18.2% (44/242)	0.06	
Body mass index, kg/m^2^	27.9±6.4 (n=344)	28.7±6.9 (n=304)	28.8±5.9 (n=263)	29.3±6.7 (n=242)	<0.001	1153
Medical history						
Current/former smoker	61.4% (207/337)	62.6% (186/297)	63.7% (163/256)	60.1% (143/238)	0.86	1128
Diabetes	58.7% (202/344)	55.9% (170/304)	53.0% (140/264)	55.4% (133/240)	0.57	1152
Prior stroke/transient ischemic attack	17.4% (59/340)	13.9% (42/303)	21.0% (55/262)	18.8% (45/239)	0.15	1144
eGFR*, mL/min per 1.73 m^2^	69.9±24.4 (n=273)	68.2±24.1 (n=246)	65.5±24.7 (n=204)	67.0±25.5 (n=183)	0.25	906
Previous anemia	24.7% (75/304)	17.8% (48/269)	14.7% (34/232)	23.0% (49/213)	0.02	1018
Peripheral vascular disease	23.8% (81/340)	20.9% (63/301)	19.8% (52/263)	24.8% (59/238)	0.46	1142
Congestive heart failure	66.2% (225/340)	61.4% (186/303)	55.5% (146/263)	56.3% (134/238)	0.03	1144
Prior MI	43.3% (141/326)	41.1% (122/297)	40.9% (105/257)	36.1% (83/230)	0.40	1110
Prior percutaneous coronary intervention	35.6% (121/340)	38.7% (117/302)	42.9% (111/259)	36.8% (88/239)	0.31	1140
Prior coronary artery bypass grafting	12.1% (41/340)	17.0% (52/305)	13.7% (36/263)	16.5% (40/242)	0.25	1150
Prior pacemaker/implantable cardioverter‐defibrillator cardiac resynchronization therapy implantation	20.3% (65/320)	17.0% (48/283)	17.4% (42/242)	12.9% (29/224)	0.17	1069
Heart failure medications						
Angiotensin‐converting enzyme inhibitor	23.9% (73/305)	21.5% (56/260)	19.5% (45/231)	19.1% (40/209)	0.51	1005
Angiotensin receptor blockers	8.5% (26/305)	12.3% (32/260)	11.3% (26/231)	11.0% (23/209)	0.51	1005
Beta blockers	60.3% (184/305)	63.8% (166/260)	60.2% (139/231)	57.9% (121/209)	0.61	1005
Echocardiography characteristics						
Left ventricular ejection fraction, %	34.4±15.2 (n=278)	34.0±15.2 (n=239)	33.2±15.3 (n=199)	36.0±15.8 (n=173)	0.34	889
Left ventricular function					0.89	889
Normal	12.9% (36/279)	13.4% (32/239)	12.1% (24/198)	11.6% (20/173)		
Mild dysfunction	24.5% (68/279)	22.2% (53/239)	23.1% (46/198)	23.1% (40/173)		
Moderate dysfunction	19.1% (53/279)	20.5% (49/239)	19.6% (39/198)	26.0% (45/173)		
Severe dysfunction	43.5% (121/279)	43.9% (105/239)	45.2% (90/198)	39.3% (68/173)		
Severe valvular disease†	11.1% (23/208)	12.3% (23/187)	9.2% (14/152)	12.8% (15/117)	0.77	664
Biological characteristics						
Leukocytes, K/uL	8.3±3.4 (n=300)	8.4±3.5 (n=269)	8.3±3.3 (n=219)	8.6±9.2 (n=201)	0.91	989
Hemoglobin, g/dL	11.7±2.0 (n=307)	11.9±2.0 (n=273)	12.4±2.2 (n=225)	12.3±2.2 (n=204)	<0.001	1009
Admission characteristics						
Angina on admission	38.7% (121/313)	45.6% (125/274)	47.3% (112/237)	44.7% (96/215)	0.18	1039
Acute MI on admission	38.1% (117/307)	36.1% (95/263)	32.5% (78/240)	34.1% (73/214)	0.56	1024
ST‐segment elevation MI	12.0% (14/117)	22.1% (21/95)	24.4% (19/78)	16.4% (12/73)	0.10	363
Out‐of‐hospital cardiac arrest	2.8% (9/318)	1.4% (4/278)	1.6% (4/239)	1.8% (4/224)	0.69	1059
Transferred from another hospital	50.2% (148/295)	47.4% (118/249)	41.1% (92/224)	33.3% (68/204)	0.001	972
Received mechanical circulatory support at another hospital	7.6% (12/158)	2.4% (3/126)	6.4% (6/94)	5.1% (4/78)	0.25	456
Inotropes/vasopressors	15.4% (47/305)	8.9% (23/260)	9.1% (21/231)	7.2% (15/209)	0.009	1005

Values are mean±SD or % (n/N). eGFR indicates estimated glomerular filtration rate; and MI, myocardial infarction.

* eGFR was calculated using 2021 Chronic Kidney Disease Epidemiology Collaboration creatinine equation.

^†^ Includes severe aortic stenosis/regurgitation and severe mitral stenosis/regurgitation.

Baseline echocardiographic characteristics are presented in Table 1. There were no significant differences between in LVEF or the prevalence of valvular disease between MBP groups. Similarly, markers of right ventricular function and other echocardiographic characteristics did not differ between MBP groups (Table S2).

### Procedural Characteristics and Outcomes

At the time of the index admission, patients with lower MBP had more frequently been transferred from another hospital compared with patients with higher MBP (*P*=0.001) (Table 1). Preprocedural inotropic agent usage was significantly higher in the lowest MBP group (*P*=0.009). There were no significant differences in other admission characteristics, including rates of angina or acute myocardial infarction presentation, cardiac arrest before admission, and placement of mechanical circulatory support before the index hospitalization between the 4 MBP groups (Table 1). During pLVAD support, blood pressure remained significantly lower in the low MBP groups (intraprocedural MBP 86±15 in the group who started with MBP ≤80 mm Hg preprocedurally versus 94±14 in those with preprocedural MBP >80 to ≤90 mm Hg versus 99±14 in those with preprocedural MBP >90 to ≤100 mm Hg versus 107±16 in those with preprocedural MBP >100 mm Hg, *P*<0.001).

In terms of procedural characteristics, the overall mean number of diseased vessels was 2.5±0.7 without differences between groups (Table 2) (*P* value=0.97). Mean pre‐PCI SYNTAX score for the entire analysis population was 28.1±12.4 (overall *P* value=0.42) and pre‐PCI mean ischemia jeopardy score was 8.9±2.1. Lower MBP groups presented with a significantly higher prevalence of left main disease (from 65.2% in those with MBP ≤80 mm Hg to 53.3% in those with MBP >100 mm Hg, *P*<0.001). There was no significant difference between MBP groups in proportion of patients who presented with graft disease (3.8%, overall *P*=0.16). At a lesion level, there were no significant differences between MBP groups in the lesion location, lesion length, Medina Classification, pre‐PCI Thrombolysis In Myocardial Infarction flow, or PCI‐related coronary complications, although lower MBP groups were more likely to have severe calcification of the target lesion compared with higher MBP groups and were less likely to have no/mild calcification of the target lesion compared with higher MBP groups (Table S3).

**Table 2:** Procedural Characteristics

	≤80 mm Hg (N=347)	>80 to ≤90 mm Hg (N=306)	>90 to ≤100 mm Hg (N=264)	>100 mm Hg (N=242)	*P* value	No.
Preprocedural hemodynamics						
MBP, mm Hg	72.7±5.8 (n=347)	85.3±2.9 (n=306)	95.0±3.0 (n=264)	109.5±7.9 (n=242)	<0.001	1159
SBP, mm Hg	104.1±12.6 (n=347)	120.3±11.5 (n=306)	133.2±12.7 (n=264)	154.8±16.8 (n=242)	<0.001	1159
DBP, mm Hg	56.9±8.3 (n=347)	67.9±6.1 (n=306)	75.9±6.4 (n=264)	86.9±10.4 (n=242)	<0.001	1159
Heart rate, bpm	75.4±15.3 (n=341)	75.9±17.4 (n=300)	76.7±16 (n=262)	78.8±19.4 (n=237)	0.09	1140
Intraprocedural hemodynamics						
MBP, mm Hg	86.3±15.3 (n=265)	94.1±14.1 (n=232)	98.7±14.2 (n=210)	106.6±15.9 (n=175)	<0.001	882
SBP, mm Hg	117.5±21.5 (n=265)	126.8±20.7 (n=232)	133.5±20.2 (n=210)	146.5±22.4 (n=176)	<0.001	883
DBP, mm Hg	70.7±15.2 (n=265)	77.8±13.8 (n=232)	81.3±14.6 (n=210)	86.6±15.9 (n=175)	<0.001	882
Angiography characteristics						
Left main disease	65.2% (223/342)	58.7% (178/303)	57.4% (151/163)	53.3% (129/242)	0.03	1150
Graft disease	2.0% (7/347)	4.6% (14/306)	3.8% (10/263)	5.4% (13/241)	0.16	1157
Number of diseased vessels	2.5±0.7	2.5±0.7	2.5±0.7	2.5±0.7	0.97	1150
1	11.6% (40/345)	10.6% (32/303)	10.0% (26/261)	11.6% (28/241)	0.99	1150
2	29.9% (103/345)	32.0% (97/303)	31.4% (82/261)	31.1% (75/241)		
3	56.2% (194/345)	56.1% (170/303)	57.5% (150/261)	56.0% (135/241)		
>3	2.3% (8/345)	1.3% (4/303)	1.2% (3/261)	1.2% (3/241)		
Synergy Between PCI with Taxus and Cardiac Surgery score	28.4±12.0 (n=237)	27.4±13.0 (n=202)	29.2±12.6 (n=181)	27.7±12.7 (n=185)	0.50	805
Ischemia jeopardy score	9.0±2.0 (n=272)	8.7±2.3 (n=235)	8.9±2.1 (n=214)	8.9±2.1 (n=203)	0.26	924
PCI procedural characteristics						
Number of vessels treated						1156
1	25.7% (89/346)	33.1% (101/305)	28.5% (75/263)	30.2% (73/242)	0.22	
2	47.7% (165/346)	42.0% (128/305)	46.0% (121/263)	43.8% (106/242)	0.50	
3	26.6% (92/346)	24.6% (75/305)	25.5% (67/263)	26.0% (63/242)	0.95	
Number of lesions treated	2.6±1.5 (n=346)	2.6±1.6 (n=305)	2.5±1.3 (n=263)	2.5±1.4 (n=242)	0.79	1156
Adjunct procedure/diagnostics	67.8% (227/335)	70.6% (207/293)	70.4% (174/247)	70.6% (163/231)	0.83	1106
Atherectomy	67.8% (154/227)	58.5% (121/207)	58.6% (102/174)	56.4% (92/163)	0.08	771
Fractional flow reserve	1.9% (4/213)	3.1% (6/192)	3.6% (6/167)	3.2% (5/155)	0.73	727
Intravascular ultrasound/optical coherence tomography	69.0% (147/213)	73.4% (141/192)	75.4% (126/167)	70.3% (109/155)	0.51	727
Temporary pacer	16.9% (36/213)	10.4% (20/192)	12.0% (20/167)	16.8% (26/155)	0.17	727
PCI arterial access						
Radial	15.4% (50/325)	17.9% (50/280)	18.1% (44/243)	15.2% (34/223)	0.72	1071
Femoral	83.4% (271/325)	80.7% (226/280)	80.7% (196/243)	83.4% (186/223)	0.72	1071
Duration of index PCI, hours	1.9 [1.3, 2.7] (n=328)	1.9 [1.2, 2.7] (n=289)	1.8 [1.2, 2.5] (n=249)	1.9 [1.3, 2.6] (n=231)	0.44	1097
Contrast volume, mL	207±100 (n=333)	206±103 (n=297)	197±100 (n=258)	211±118 (n=239)	0.48	1127
Impella characteristics						
Device used						
Impella 2.5	31.5% (109/346)	27.1% (83/306)	31.1% (82/264)	29.3% (71/242)	0.63	1158
Impella CP	68.5% (237/346)	72.9% (223/306)	68.9% (182/264)	70.7% (171/242)	0.63	1158
Impella access						
Femoral	92.8% (322/347)	95.4% (292/306)	96.6% (255/264)	93.4% (226/242)	0.16	1159
Subclavian/transaxillary/transcaval	7.2% (25/347)	4.6% (14/306)	3.4% (9/264)	6.6% (16/242)	0.15	1159
Duration of percutaneous left ventricular assist device support, h	1.6 [1.1, 2.5] (n=307)	1.6 [1.0, 2.6] (n=268)	1.4 [1.0, 2.1] (n=233)	1.5 [1.0, 2.3] (n=208)	0.10	1016

Values are mean±SD, median [Q1, Q3], or n (%). Time‐based variables are reported as median [interquartile range, denominator]. DBP indicates diastolic blood pressure; MBP, mean blood pressure; PCI, percutaneous coronary intervention; and SBP, systolic blood pressure.

There were no differences between groups in duration of PCI, duration of pLVAD‐support, type of mechanical circulatory support device used, number of vessels treated, radial/femoral PCI arterial access, or vascular access sites for Impella. Regarding adjunct procedures, there was a trend toward more frequent use of atherectomy in the lower MBP groups (from 67.8% in those with MBP ≤80 mm Hg to 56.4% in those with MBP >100 mm Hg, *P*=0.08), but there was no significant difference in use of fractional flow reserve, intravascular ultrasound/optical coherence tomography, or temporary pacemaker usage across groups (Table 2).

### In‐Hospital Outcome

At the time of discharge, there was a gradient in mortality according to MBP, but this difference was not significant (*P*=0.18) (Table 3). Furthermore, there were no significant variations in terms of in‐hospital adverse events, such as ventricular arrhythmia, vascular/cardiac complications requiring surgery, vascular complications, limb ischemia, respiratory failure, and acute renal dysfunction (stage 2 or 3). Similarly, no significant disparities were found in the rates of intensive care unit admission or the duration of hospitalization. Although patients in the lower MBP groups were more likely to require a blood transfusion during their hospital stay, there were no significant differences observed in overall major bleeding rates.

**Table 3:** In‐Hospital Adverse Events

	≤80 mm Hg (N=347)	>80 to ≤90 mm Hg (N=306)	>90 to ≤100 mm Hg (N=264)	>100 mm Hg (N=242)	*P* value
In‐hospital adverse events					
Death	20 (5.8)	13 (4.3)	8 (3.0)	6 (2.5)	0.18
Hypotensive episode	18 (5.2)	6 (2.0)	7 (2.7)	11 (4.6)	0.10
Cardiogenic shock	16 (4.6)	7 (2.3)	4 (1.5)	4 (1.7)	0.06
Cardiac arrest	13 (3.8)	3 (1.0)	6 (2.3)	3 (1.2)	0.07
Ventricular arrhythmia	5 (1.5)	5 (1.6)	6 (2.3)	3 (1.2)	0.84
Vascular/cardiac complication requiring surgery	9 (2.6)	3 (1.0)	6 (2.3)	1 (0.4)	0.12
Vascular complication not requiring surgery	6 (1.7)	6 (1.96)	3 (1.1)	3 (1.2)	0.86
Limb ischemia	5 (1.5)	7 (2.29)	6 (2.3)	2 (0.8)	0.51
Major bleeding (Bleeding Academic Research Consortium ≥3a)	9 (2.6)	6 (2.0)	8 (3.0)	7 (2.9)	0.86
Anemia requiring transfusion	32 (9.3)	33 (10.8)	12 (4.6)	16 (6.6)	0.03
Respiratory failure	10 (2.9)	4 (1.31)	2 (0.8)	3 (1.2)	0.21
Acute renal dysfunction (stage 2 or 3)	20 (5.8)	16 (5.2)	8 (3.0)	10 (4.1)	0.40
Procedural/admission end points					
Change in Synergy between PCI with Taxus and Cardiac Surgery score	−22.54 (10.5)	−20.34 (10.5)	−22.03 (11.4)	−20.51 (10.4)	0.09
Change in ischemia jeopardy score	−7.19 (2.26)	−6.66 (2.55)	−6.83 (2.45)	−6.94 (2.55)	0.11
Duration of hospitalization, d	7.0 [3.0–12.5]	6.5 [3.0–10.0]	5.0 [2.0–10.0]	5.0 [1.0–8.0]	0.29
Intensive care unit admission	189 (54.5)	154 (50.3)	118 (44.7)	133 (55.0)	0.06

Values for in‐hospital events are median [Q1, Q3], or n (%).

The group with the lowest preprocedural MBP presented with a higher prevalence of anemia, required more frequent usage of inotropic agents (Table 1), and exhibited increased use of atherectomy, greater number of revascularized lesions, and longer interventional procedure time (Table 2). In this context, they experienced more frequent complications, including a higher incidence of cardiorespiratory arrest, hypotensive episodes, and longer intensive care unit hospitalization (Table 3).

### Major Adverse Cardiovascular and Cerebrovascular Events

**Figure 1:**
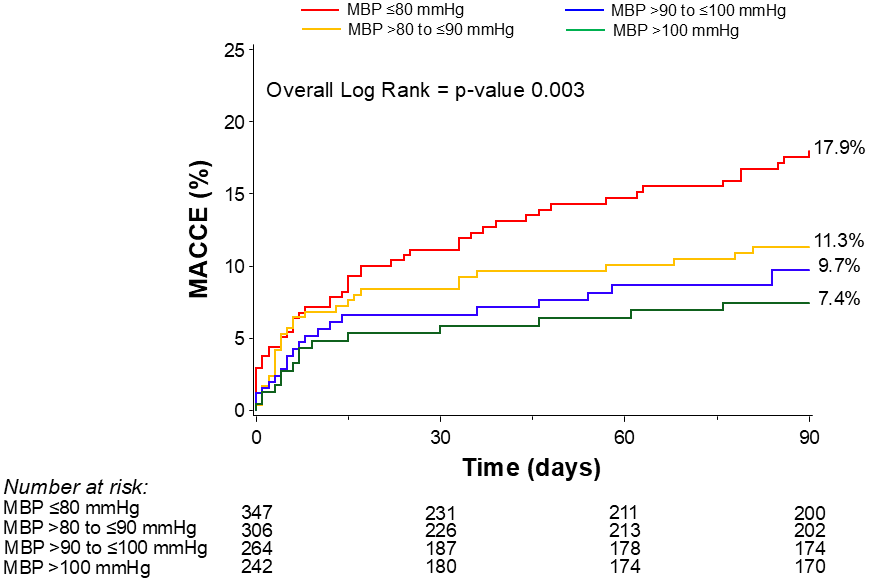
Kaplan–Meier curves for 90‐day major adverse cardiovascular and cerebrovascular events stratified by mean blood pressure. MACCE indicates major adverse cardiovascular and cerebrovascular events; and MBP, mean blood pressure. MACCE is defined as the composite of all‐cause death, myocardial infarction, stroke/transient ischemic attack, and repeat revascularization.

**Figure 2:**
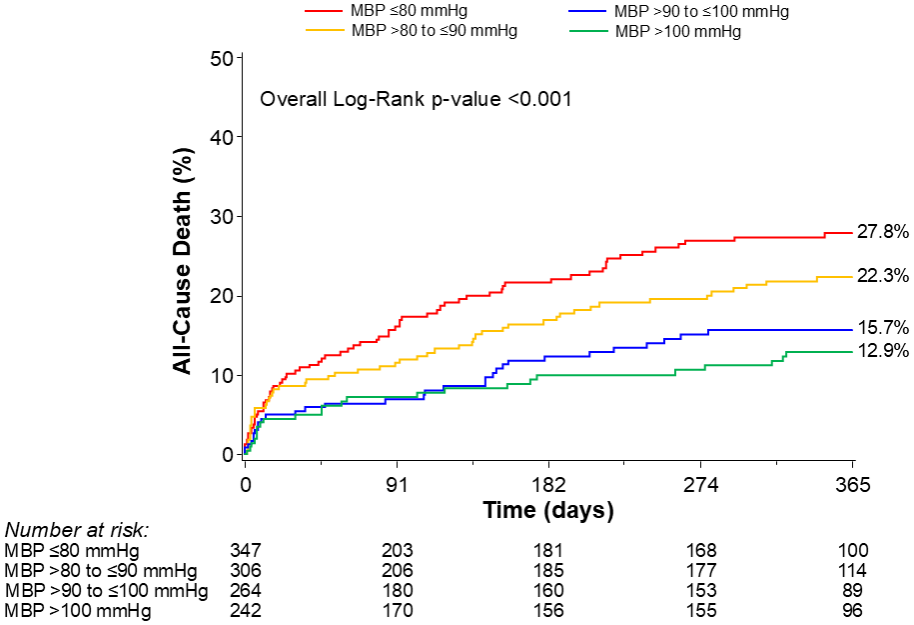
Kaplan–Meier curves for 1‐year mortality stratified by mean blood pressure. MBP indicates mean blood pressure.

**Figure 3:**
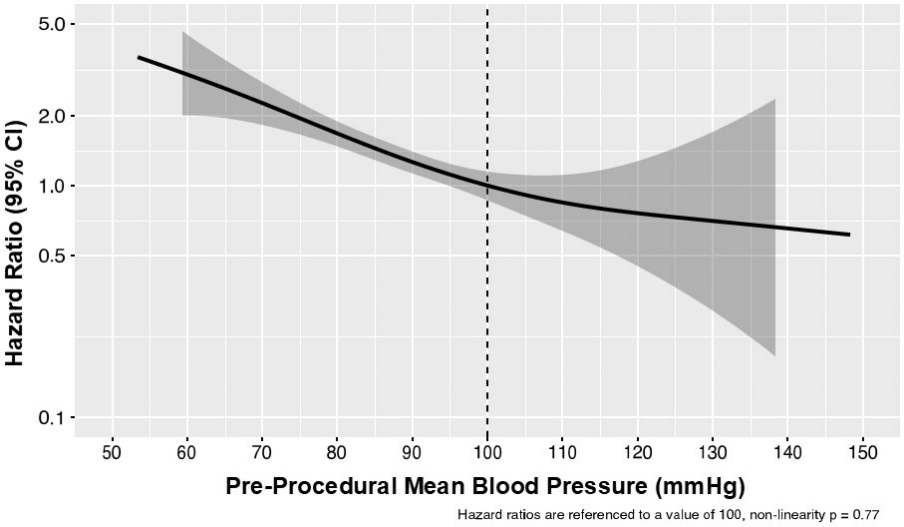
Association of preprocedural blood pressure with 1‐year mortality. Spline for hazard ratio.

At 90 days, there were significant differences in the rates of MACCE between groups, with a notable increase in MACCE seen in the lowest MBP group (from 17.9% in those with MBP ≤80 mm Hg to 7.4% in those with MBP >100 mm Hg, *P*=0.003) (Table 4, Figure 1). The difference in MACCE was driven mainly by differences in all‐cause death (from 15.5% in those with MBP ≤80 mm Hg to 7.1% in those with MBP >100 mm Hg, *P*=0.005). There were no significant differences between MBP groups for the other components of MACCE, namely myocardial infarction (*P*=0.10), stroke/transient ischemic attack (*P*=0.80), and repeat revascularization (0.60) (Figure S3). All‐cause death at 1 year was also significantly higher in the lower MBP groups (ranging from 27.8% in the lowest MBP group to 12.9% in the highest, *P*<0.001) (Table 4, Figure 2). The relationship between MBP and all‐cause mortality appeared to be linear, as indicated by the HR spline representation (Figure 3), with a plateau of benefit observed above approximately 110 mm Hg MBP.

**Table 4:** Adverse Events at 90 Days and 1 Year

	≤80 mm Hg (N=347)	>80 to ≤90 mm Hg (N=306)	>90 to ≤100 mm Hg (N=264)	>100 mm Hg (N=242)	*P* value
90‐d MACCE*	17.9% (51)	11.3% (30)	9.7% (15)	7.4% (15)	0.003
Death	15.5% (43)	9.9% (26)	6.5% (14)	7.1% (14)	0.005
Myocardial infarction	5.1% (13)	2.8% (7)	4.4% (2)	1.0% (2)	0.104
Stroke/transient ischemic attack	1.4% (5)	1.1% (3)	2.3% (3)	1.3% (3)	0.802
Repeat revascularization	3.0% (7)	2.1% (5)	2.1% (2)	1.1% (2)	0.601
1‐y all‐cause death	27.8% (72)	22.2% (54)	15.7% (31)	12.9% (24)	<0.001

Values for MACCE and 1‐y all‐cause death are presented as Kaplan–Meier rates (number of events). MACCE indicates major adverse cardiac and cerebrovascular events.

* MACCE is defined as the composite of all‐cause death, myocardial infarction, stoke/transient ischemic attack, and repeat revascularization.

When using univariate Cox regression, preprocedural MBP was significantly associated with 1‐year mortality (HR, 0.78 [95% CI, 0.70–0.87] per 10 mm Hg increment, *P*<0.001) (Table S4). The association between preprocedural MBP and 1‐year mortality remained statistically significant in the multivariable Cox model (HR, 0.79, [95% CI, 0.71–0.88] per 10 mm Hg increment, *P*<0.001), with adjustments for other factors such as age, previous stroke, acute myocardial infarction on admission, hemoglobin levels, estimated glomerular filtration rate, LVEF, left main disease, and initial SYNTAX score (Table S5). The association of preprocedural MBP with mortality remains significant in multivariate analysis as well as with the addition of preprocedural inotropes/vasopressors as adjustment variables (Table S6), or with the addition of body mass index and race (Table S7).

### Hypotensive Episodes

Although 44 patients experienced intraprocedural hypotensive episodes, there was no statistically significant difference between MBP groups (*P*=0.10). Patients who experienced hypotensive episodes during the procedure were found to have a significantly higher incidence of chronic kidney disease, graft disease, a higher number of overall lesions, and no other differences in other baseline characteristics (Table S8). On multivariable logistic regression, several factors were found to be significantly associated with the occurrence of hypotensive episodes (Table S9). These factors included a history of chronic kidney disease, the admission with an acute myocardial infarction, angiographically determined graft disease and the number of diseased vessels, and finally, nonfemoral access for the Impella device (ie, subclavian, transaxillary, or transcaval access).

## DISCUSSION

This study is the first to comprehensively examine the association of preprocedural hemodynamic profiles, including MBP, and the procedural, postprocedural, and longer‐term outcomes in patients undergoing pLVAD‐supported HRPCI. The main findings are (1) low preprocedural MBP, but not intraprocedural MBP, was associated with adverse events; (2) with pLVAD support, intraprocedural hemodynamic stability was high, and hypotensive episodes were infrequent and not related to preprocedural MBP; (3) there was a graded association between preprocedural MBP and 1‐year mortality, which plateaued at an MBP of ~110 mm Hg; and (4) clinically defined groups with 10 mm Hg increments in MBP allowed for differential risk stratification at multiple follow‐up times.

From a global perspective, MBP is regulated by numerous, complex, central, and peripheral mechanisms. MBP determines organ perfusion and, in this regard, serves as an indicator of hemodynamic statues. MBP is a strong predictor of outcomes in patients undergoing non‐HRPCI.^9^ In the context of pLVAD supported HRPCI, MBP assumes notability, as the goal of the mechanical circulatory support in this setting is to prevent intraprocedural hypotension and to stabilize hemodynamics. Beyond the immediate effect, this initial hemodynamic assessment enables risk stratification for short‐, medium‐, and long‐term mortality.

### Immediate Impact of Preprocedural Blood Pressure

In our study, we found that a low preprocedural MBP was associated with an increasing use of inotropes, presence of anemia, and a higher occurrence of lesions in the left main coronary artery despite similar LVEF, similar SYNTAX score, and similar number of significant lesions. In the setting of pLVAD support, rates of intraprocedural hypotension were low and similar across baseline MBP levels. In addition, it is noteworthy that most procedural characteristics including completeness of revascularization were similar. The observed consistency in terms of revascularization could potentially be attributed to the use of pVAD support.

### Safety of High‐Risk PCI and Prevention of Hypotensive Episodes

Hypotension can lead to adverse outcomes for patients and compromise the success of the PCI. These data demonstrated a high level of safety and effectiveness in preventing hypotensive episodes through temporary pLVAD support, with <5% of episodes occurring in this HRPCI population and no association between preprocedural MBP and hypotensive episodes during the procedure. It is important to note that, although intraprocedural MBP remained lower in the lower preprocedural MBP groups, intraprocedural MBP was not associated with outcomes, further underscoring the effectiveness of mitigating the adverse effects of hemodynamic compromise during the procedure.

### Short‐Term Impact of Preprocedural Blood Pressure

Despite the fact that patients with overt cardiogenic shock were not included in the registry, these findings imply that stratification by preprocedural MBP identifies patients hemodynamically bordering on the realm of cardiogenic shock. These differences translated to a very early divergence in Kaplan–Meier curves for all‐cause mortality, particularly during the initial weeks including the index hospitalization, between the low MBP and higher MBP groups with a cutoff of 90 mm Hg (≤90 mm Hg versus >90 mm Hg). Among patients with a preprocedural MBP <90 mm Hg, MBP categories further stratified risk within the first month following hospitalization (≤80 mm Hg versus >80 to ≤90 mm Hg). The group at highest risk (≤80 mm Hg) presented more comorbid conditions, more complex procedural characteristics, worse hemodynamic status, and worse outcomes. Notably, these differences persisted despite similar LVEF and comparable pre‐PCI SYNTAX scores in the >80 to ≤90 mm Hg group. This highlights the importance of the individualization of PCI procedures, as those with a MBP<90 mm Hg were in a very high‐risk category, hemodynamically bordering on the realm of cardiogenic shock, having highly complex coronary lesions, with multiple comorbidities patients, thus constituting a “triple” high‐risk PCI situation.

### Long‐Term Impact of Preprocedural Blood Pressure

One of the most striking findings of our study was the strong, continuous relationship between preprocedural MBP and 1‐year mortality—an association that remained significant even after adjusting for differences in other characteristics between the MBP groups. There was a noticeable separation of the Kaplan–Meier curves between the low‐risk groups (>90 to ≤100 mm Hg versus >100 mm Hg group) after 5 months of follow‐up. The influence of preprocedural blood pressure on long‐term outcomes after PCI has previously been documented in the context of stable patients with noncomplex procedures^9^ or acute myocardial infarction.^13^

### Limitations

This study has several limitations that must be considered. It is important to note that this analysis is post hoc and the observed associations between MBP and clinical outcomes may be confounded. The methodology does not allow for the definitive determination of an optimal target for MBP to initiate a high‐risk PCI procedure. The assessment of MBP was conducted at a single point at the beginning of the procedure and did not consider the dynamic changes during the procedure. Additional analyses that consider the dynamic nature of MBP could provide an additional insight in the future.

## CONCLUSIONS

This study demonstrates the impact of preprocedural hemodynamic profiles, including MBP, on 1‐year mortality and emphasizes the importance of considering these profiles as potential predictors of long‐term outcomes in patients. This association stemmed from differences in baseline clinical characteristics rather than hemodynamic instability experienced during the procedures but remained independently prognostic even after adjustment for these baseline differences. Furthermore, procedural hemodynamic stability was high, even in the presence of challenging hemodynamic conditions.

## Sources of Funding

The PROTECT III study, as part of The Global cVAD study, was sponsored by Abiomed Inc. (Danvers, MA, USA).

## Disclosures

A. J. Lansky received speaker fees from Keystone Heart. B. Redfors received consultant fees from Pfizer and Boehringer Ingelheim. D. Burkhoff has received institutional educational grant support from Abiomed; and institutional grant support from Ancora Heart. D. J. Cohen has received research grant support from Edwards Lifesciences, Abbott, Boston Scientific, Corvia Medical, Philips, Brain‐Q, Saranas, Zoll Medical, CathWorks, and ANCORA, and has received consultant fees from Medtronic, Edwards Lifesciences, Abbott, and HeartBeam. M. B. Basir has been a consultant/speaker for Abiomed, Boston Scientific, Chiesi, Saranas, and Zoll. W. W. O'Neill reports grant/research support from St. Jude Medical, Edwards Life Sciences, and Biomed; consulting fees/honoraria from Medtronic and Abiomed; and major stock shareholder/equity in Synecor, Accumed, Neovasc, Tendyne, and Mitral Align. J. F. Granada is the co‐founder of Cephea Valve Technologies (Abbott) and is chief executive officer of Cardiovascular Research Foundation.

## Supporting information

Tables S1–S9
Figures S1–S3
