# Supplementary material for: Impact of Mean Blood Pressure Profiles in Percutaneous Left Ventricular Assist Device‐Supported High‐Risk Percutaneous Coronary Intervention: The PROTECT III Study
Source: J Am Heart Assoc. 2025 May 15;14(10):e036367. doi: 10.1161/JAHA.124.036367 (PMC12184586; doi:10.1161/JAHA.124.036367)
Supplement: Supplementary file 1 — Tables S1–S9 Figures S1–S3 [file JAH3-14-e036367-s001.pdf]

Supplemental Appendix

Supplemental Table S1. Baseline Characteristics of Included and Excluded Patient Population.

	Included (n=1159)	Excluded (n=78)	p Value
Demographics			
Age, years	71.1 ± 11.1 (n=1159)	70.4 ± 11.0 (n=78)	0.61
Sex, male	73.5% (852/1159)	67.9% (53/78)	0.35
Race			
White or Caucasian	66.7% (773/1159)	74.4% (58/78)	0.20
Black or African American	12.9% (149/1159)	5.1% (4/78)	0.07
Asian	3.2% (37/1159)	1.3% (1/78)	0.51
American Indian or Alaska native	0.5% (6/1159)	0% (0/78)	1.00
Native Hawaiian or Other Pacific Islander	0.1% (1/1159)	0% (0/78)	1.00
Other race	3.5% (41/1159)	1.3% (1/78)	0.51
Unknown race	13.1% (152/1159)	17.9% (14/78)	0.30
Body Mass Index, kg/m^2	28.6 ± 6.5 (n=1153)	29.1 ± 6.1 (n=78)	0.55
Medical History			
Current/Former Smoker	62.0% (699/1128)	64.9% (50/77)	0.69
Diabetes mellitus	56.0% (645/1152)	55.8% (43/77)	1.00
Stroke/transient ischemic attack	17.6% (201/1144)	10.3% (8/78)	0.13
Chronic kidney disease	32.2% (369/1145)	25.6% (20/78)	0.28
eGFR*, mL/min/1.73 m^2	67.9 ± 24.6 (n=906)	71.9 ± 23.9 (n=54)	0.24
Anemia	20.2% (206/1018)	12.3% (8/65)	0.16
Peripheral vascular disease	22.3% (255/1142)	20.0% (15/75)	0.74
Congestive heart failure	60.4% (691/1144)	59.7% (46/77)	1.00
Prior myocardial infarction	40.6% (451/1110)	37.3% (28/75)	0.65
Prior percutaneous coronary intervention	38.3% (437/1140)	40.3% (31/77)	0.83
Prior coronary artery bypass grafting	14.7% (169/1150)	14.1% (11/78)	1.00
Prior aortic/mitral valve intervention	2.5% (27/1061)	4.3% (3/70)	0.43
Prior PM/ICD/CRT implantation	17.2% (184/1069)	17.1% (12 /70)	1.00
Echocardiography Characteristics			
Left ventricular ejection fraction, %	34.3 (15.4) (n=889)	33.1 (14.9) (n=51)	0.56
Severe valvular disease*	11.3% (75/664)	5.4% (2/37)	0.42
Biological Characteristics			
Leukocytes (K/uL)	8.4 (5.1) (n=989)	8.7 (3.7) (n=62)	0.61
Hemoglobin (g/dL)	12.0 (2.1) (n=1009)	12.1 (2.3) (n=63)	0.82
Angiography Characteristics			
LM disease	59.2% (681/1150)	49.4% (38/77)	0.11
Graft disease	3.8% (44/1157)	5.2% (4/77)	0.54
Number of Diseased Vessels	2.5 (0.7) (n=1150)	2.5 (0.8) (n=76)	0.76
1	10.9% (126/1159)	15.4% (12/78)	0.30
2	30.8% (357/1159)	24.4% (19/78)	0.28
3	56.0% (649/1159)	55.1% (43/78)	0.98
>3	1.6% (18/1150)	2.6% (2/76)	0.31
SYNTAX score	28.2 ± 12.5 (n=805)	25.9 ± 10.7 (n=45)	0.21
Ischemia Jeopardy Score	8.9 ± 2.1 (n=924)	8.4 ± 2.2 (n=51)	0.12

Data presented as n (%) or mean (standard deviation), where applicable. *eGFR was calculated using 2021 CKD-EPI Creatinine Equation. †Includes severe aortic stenosis/regurgitation and severe mitral stenosis/regurgitation. CRT denotes cardiac resynchronization therapy; eGFR, estimated glomerular filtration rate; ICD, implantable cardioverter-defibrillator; LM, left main; PCI, percutaneous coronary intervention; PM, pacemaker; SYNTAX, the Synergy between PCI with Taxus and Cardiac Surgery.

Supplemental Table S2. Echocardiographic Characteristics Stratified by Mean Blood Pressure.

	≤80 mmHg (N=347)	>80 to ≤90 mmHg (N=306)	>90 to ≤100 mmHg (N=264)	>100 mmHg (N=242)	p Value	N
Right ventricular function						530
Normal	71.7% (124/173)	67.3% (99/147)	66.7% (76/114)	72.9% (70/96)	0.64	
Mild dysfunction	16.8% (29/173)	17.7% (26/147)	21.9% (25/114)	14.6% (14/96)	0.54	
Moderate dysfunction	8.1% (14/173)	13.6% (20/147)	8.8% (10/114)	8.3% (8/96)	0.34	
Severe dysfunction	3.5% (6/173)	1.4% (2/147)	2.6% (3/114)	4.2% (4/96)	0.55	
Mitral valve regurgitation						644
No/Trace	22.0% (45/205)	23.6% (42/178)	29.3% (44/150)	18.0% (20/111)	0.17	
Mild	45.9% (94/205)	48.9% (87/178)	45.3% (68/150)	45.0% (50/111)	0.89	
Moderate	25.4% (52/205)	23.6% (42/178)	22.0% (33/150)	32.4% (36/111)	0.25	
Severe	6.8% (14/205)	3.9% (7/178)	3.3% (5/150)	4.5% (5/111)	0.41	
Mitral valve stenosis						464
No/Trace	92.1% (129/140)	89.8% (115/128)	92.6% (100/108)	88.6% (78/88)	0.72	
Mild	6.4% (9/140)	6.3% (8/128)	5.6% (6/108)	6.8% (6/88)	0.99	
Moderate	1.4% (2/140)	3.9% (5/128)	0.9% (1/108)	3.4% (3/88)	0.37	
Severe	0% (0/140)	0% (0/128)	0.9% (1/108)	1.1% (1/88)	0.25	
Aortic valve regurgitation						598
No/Trace	71.8% (135/188)	69.8% (120/172)	74.6% (100/134)	73.1% (76/104)	0.81	
Mild	21.8% (41/188)	23.8% (41/172)	21.6% (29/134)	17.3% (18/104)	0.65	
Moderate	5.9% (11/188)	6.4% (11/172)	3.7% (5/134)	9.6% (10/104)	0.32	
Severe	0.5% (1/188)	0% (0/172)	0% (0/134)	0% (0/104)	1.00	
Aortic valve stenosis						563
No/Trace	86.4% (152/176)	80.5% (132/164)	79.8% (99/124)	82.8% (82/99)	0.41	
Mild	5.1% (9/176)	4.9% (8/164)	8.1% (10/124)	4.0% (4/99)	0.54	
Moderate	3.4% (6/176)	4.9% (8/164)	5.7% (7/124)	3.0% (3/99)	0.72	
Severe	5.1% (9/176)	9.8% (16/164)	6.5% (8/124)	10.1% (10/99)	0.29	
Pulmonary valve regurgitation						190
Absent	50% (30/60)	54.1% (33/61)	50% (18/36)	54.5% 18/33	0.95	
Mild	40% (24/60)	42.6% (26/61)	38.9% (14/36)	45.5% 15/33	0.94	
Moderate	10% (6/60)	3.3% (2/61)	11.1% (4/36)	0% 0/33	0.10	
Severe	0% (0/60)	0% (0/61)	0% (0/36)	0% 0/33	-	
Tricuspid valve regurgitation						222
Absent	22.9% (16/70)	29.7% (22/74)	30% (12/40)	28.9% (11/38)	0.78	
Mild	58.6% (41/70)	51.4% (38/74)	60% (24/40)	55.3% (21/38)	0.78	
Moderate	15.7% (11/70)	14.9% (11/74)	7.5% (3/40)	13.2% (5/38)	0.65	
Severe	2.9% (2/70)	4.1% (3/74)	2.5% (1/40)	2.6% (1/38)	0.96	

Data presented as n/N (%).

Supplemental Table S3. Angiographic and Procedural Data: Lesion-Level Analysis.

	≤80 mmHg (N=798)	>80 to ≤90 mmHg (N=656)	>90 to ≤100 mmHg (N=605)	>100mmHg (N=565)	p Value	N
**Lesion location**						2583
Proximal	29.0% (227/784)	30.0% (193/643)	30.1% (180/599)	30.2% (168/557)	0.95	
Middle	18.5% (145/784)	19.0% (122/643)	16.7% (100/599)	18.9% (105/557)	0.72	
Distal	17.0% (133/784)	18.2% (117/643)	19.5% (117/599)	17.2% (96/557)	0.62	
Ostial	35.6% (279/784)	32.8% (211/643)	33.7% (202/599)	33.8% (188/557)	0.73	
**Degree of Calcification**						2533
None/Mild	30.5% (239/783)	31.9% (199/624)	34.2% (199/582)	40.3% (219/544)	0.002	
Moderate	18.4% (144/783)	16.2% (101/624)	15.5% (90/582)	18.2% (99/544)	0.42	
Severe	51.1% (400/783)	51.9% (324/624)	50.3% (293/582)	41.5% (226/544)	0.001	
**Lesion Length (mm)**	14.1 ± 12.1 n=757	14.5 ± 13.6 n=615	13.4 ± 11.0 n=585	14.8 ± 11.9 n=533	0.21	2490
**Pre-PCI TIMI**						2551
0	6.3% (49/780)	4.8% (30/621)	4.5% (27/599)	7.1% (39/551)	0.20	
1	2.3% (18/780)	1.1% (7/621)	1.3% (8/599)	2.4% (13/551)	0.22	
2	3.2% (25/780)	2.7% (17/621)	2.2% (13/599)	1.3% (7/551)	0.14	
3	88.2% (688/780)	91.3% (567/621)	92.0% (551/599)	89.3% (492/551)	0.07	
**Medina Classification**						1671
1,1,1	39.1% (196/501)	38.5% (157/408)	41.4% (171/413)	39.0% (136/349)	0.83	
1,1,0	16.4% (82/501)	15.2% (62/408)	16.9% (70/413)	17.8% (62/349)	0.81	
1,0,1	8.0% (40/501)	9.1% (37/408)	10.4% (43/413)	8.3% (29/349)	0.61	
0,1,1	13.2% (66/501)	15.4% (63/408)	13.8% (57/413)	12.9% (45/349)	0.72	
1,0,0	7.6% (38/501)	6.6% (27/408)	5.6% (23/413)	8.0% (28/349)	0.53	
0,1,0	11.6% (58/501)	11.8% (48/408)	9.2% (38/413)	12.0% (42/349)	0.55	
0,0,1	4.2% (21/501)	3.4% (14/408)	2.7% (11/413)	2.0% (7/349)	0.30	
**Post-PCI TIMI**						2577
0	0.4% (3/778)	0.5% (3/646)	0.1% (1/600)	1.2% (7/553)	0.09	
1	0.3% (2/778)	0% (0/646)	0% (0/600)	0.4% (2/553)	0.24	
2	0.6% (5/778)	0% (0/646)	0.7% (4/600)	1.1% (6/553)	0.05	
3	98.7% (768/778)	99.5% (643/646)	99.2% (595/600)	97.3% (538/553)	0.004	
**Composite of PCI related coronary complications***	3.6% (28/780)	2.2% (14/647)	1.7% (10/599)	3.1% (17/557)	0.12	2583

Data presented as % (n/N) or mean ± standard deviation, where applicable. *includes no reflow, abrupt closure, dissection, distal embolus, perforation. PCI denotes percutaneous coronary intervention; TIMI, thrombolysis in myocardial infarction.

Supplemental Table S4. Univariate Cox Analysis for 1-year death

	HR (95% CI)	p Value
*Pre-procedural**		
**Systolic blood pressure** (mmHg)	0.88 [0.82;0.94]	<0.001
**Diastolic blood pressure** (mmHg)	0.79 [0.71;0.88]	<0.001
**Mean blood pressure** (mmHg)	0.78 [0.70;0.87]	<0.001
*During Procedure**		
**Systolic blood pressure** (mmHg)	0.92 [0.85;0.99]	0.018
**Diastolic blood pressure** (mmHg)	0.94 [0.85;1.05]	0.271
**Mean blood pressure** (mmHg)	0.91 [0.82;1.01]	0.067
*Post-Procedural**		
**Systolic blood pressure** (mmHg)	0.94 [0.88;1.01]	0.083
**Diastolic blood pressure** (mmHg)	0.91 [0.82;1.00]	0.062
**Mean blood pressure** (mmHg)	0.90 [0.82;1.00]	0.041

**per 10 mmHg increment*

Supplemental Table S5. Multivariable models for 1-year death

**N=1,237**	**HR (95% CI)**	**p Value**
Medical history		
**Age** (years) *per 10 years increment*	1.19 (1.03, 1.37)	0.02
**Previous Stroke**	1.50 (1.07, 2.10)	0.02
Admission Characteristics		
**Acute MI on admission**	1.45 (1.08, 1.96)	0.01
Biological parameters		
**Hemoglobin** (g/dL)	0.89 (0.82, 0.95)	0.001
**eGFR** (mL/min/1.73 m^2), *per 10 UI increment*	0.93 (0.87, 0.99)	0.04
Echocardiography parameters		
**Left ventricular ejection fraction** (%)	0.82 (0.73, 0.91)	<0.001
Angiography Characteristics		
**Left main disease**	1.38 (0.99, 1.92)	0.06
**SYNTAX score**	1.01 (0.99, 1.02)	0.36
Preprocedural Parameters		
**Mean blood pressure** (mmHg) *per 10 mmHg increment*	0.79 (0.71, 0.88)	<0.001

eGFR denotes estimated glomerular filtration rate; MI, myocardial infarction; SYNTAX, the Synergy between PCI with Taxus and Cardiac Surgery

Supplemental Table S6. Multivariable models for 1-year death including Inotropes/Vasopressors

**N=1,237**	**HR (95% CI)**	**p Value**
Medical history		
**Age** (years) *per 10 years increment*	1.34 (1.10, 1.64)	0.004
**Previous Stroke**	1.20 (0.73, 1.99)	0.47
Admission Characteristics		
**Acute MI on admission**	1.46 (0.99, 2.16)	0.05
Biological parameters		
**Hemoglobin** (g/dL)	0.90 (0.82, 1.00)	0.05
**eGFR** (mL/min/1.73 m^2), *per 10 UI increment*	0.96 (0.87, 1.06)	0.41
Echocardiography parameters		
**Left ventricular ejection fraction** (%)	0.76 (0.66, 0.87)	<0.001
Angiography Characteristics		
**Left main disease**	1.18 (0.78, 1.79)	0.44
**SYNTAX score**	1.01 (0.99, 1.02)	0.39
Preprocedural Parameters		
**Mean blood pressure** (mmHg) *per 10 mmHg increment*	0.83 (0.71, 0.98)	0.02
Medication		
**Inotropes/vasopressors**	0.87 (0.47, 1.61)	0.67

eGFR denotes estimated glomerular filtration rate; MI, myocardial infarction; SYNTAX, the Synergy between PCI with Taxus and Cardiac Surgery

Supplemental Table S7. Multivariable models for 1-year death including Ethnicity and BMI

N=1,237	HR (95% CI)	p Value
Medical history		
**Age** (years) *per 10 years increment*	1.38 (1.11, 1.71)	0.004
**Previous Stroke**	1.12 (0.68, 1.85)	0.65
**Race : Black or African American**	1.33 (0.75, 2.38)	0.33
**Body Mass Index, kg/m^2**	0.99 (0.96, 1.02)	0.63
Admission Characteristics		
**Acute MI on admission**	1.39 (0.95, 2.03)	0.08
Biological parameters		
**Hemoglobin** (g/dL)	0.91 (0.82, 1.00)	0.05
**eGFR** (mL/min/1.73 m^2), *per 10 UI increment*	0.96 (0.87, 1.05)	0.37
Echocardiography parameters		
**Left ventricular ejection fraction** (%)	0.78 (0.68, 0.89)	<0.001
Angiography Characteristics		
**Left main disease**	1.22 (0.81, 1.83)	0.34
**SYNTAX score**	1.01 (0.99, 1.02)	0.33
Preprocedural Parameters		
**Mean blood pressure** (mmHg) *per 10 mmHg increment*	0.83 (0.71, 0.96)	0.02

eGFR denotes estimated glomerular filtration rate; MI, myocardial infarction; SYNTAX, the Synergy between PCI with Taxus and Cardiac Surgery

Supplemental Table S8. Baseline Characteristics of Patients Presenting Hypotensive Episode

	No Hypotensive episode (n=1192)	Hypotensive episode (n=44)	p Value	N
Demographics				
Age, years	71.0 ± 11.1 (n=1192)	71.3 ± 10.4 (n=44)	0.85	1236
Sex, male	73.2% (872/1192)	75.0% (33/44)	0.92	1236
Race				
White or Caucasian	67.4% (804/1192)	59.1% (26/44)	0.32	1236
Black or African American	12.2% (146/1192)	15.9% (7/44)	0.62	1236
Asian	3.1% (37/1192)	2.3% (1/44)	1.00	1236
American Indian or Alaska native	0.50% (6/1192)	0% (0/44)	1.00	1236
Native Hawaiian or Other Pacific Islander	0.08% (1/1192)	0% (0/44)	1.00	1236
Other race	3.4% (40/1192)	4.6% (2/44)	0.66	1236
Unknown race	13.3% (158/1192)	18.2% (8/44)	0.48	1236
Body Mass Index, kg/m^2	28.6 ± 6.4 (n=1188)	29.4 ± 8.1 (n=42)	0.56	1230
Medical History				
Current/Former Smoker	62.2% (721/1160)	63.6% (28/44)	0.97	1204
Diabetes mellitus	55.4% (657/1185)	69.8% (30/43)	0.09	1228
Stroke/transient ischemic attack	17.3% (204/1179)	11.6% (5/43)	0.45	1222
Chronic kidney disease	31.2% (368/1180)	47.6% (20/42)	0.04	1222
eGFR*, mL/min/1.73 m^2	68.1 ± 24.6 (n=926)	69.3 ± 24.6 (n=33)	0.78	959
Anemia	19.7% (208/1058)	24.0% (6/25)	0.61	1083
Peripheral vascular disease	22.1% (259/1173)	23.3% (10/43)	1.00	1216
Congestive heart failure	60.4% (711/1177)	58.1% (25/43)	0.89	1220
Prior myocardial infarction	40.3% (460/1142)	42.9% (18/42)	0.86	1184
Prior PCI	38.7% (454/1172)	29.5% (13/44)	0.28	1216
Prior coronary artery bypass grafting	14.3% (169/1184)	25.0% (11/44)	0.08	1228
Prior aortic/mitral valve intervention	2.72% (30/1104)	0% (0/27)	1.00	1131
Prior PM/ICD/CRT implantation	17.3% (192/1111)	14.8% (4/27)	1.00	1138
Echocardiography Characteristics				
Left ventricular ejection fraction, %	34.3 (15.4) (n=906)	33.4 (13.1) (n=34)	0.69	940
Severe valvular disease†	10.8% (73/676)	16.0% (4/25)	0.34	701
Biological Characteristics				
Leukocytes (K/uL)	8.35 (5.03) (n=1011)	9.77 (4.88) (n=39)	0.082	1050
Hemoglobin (g/dL)	12.1 (2.12) (n=1032)	11.6 (2.30) (n=39)	0.247	1071
Angiography Characteristics				
LM disease	58.8% (696/1183)	23 (52.3)	0.48	1227
Graft disease	3.5% (42/1189)	13.6% (6/42)	0.006	1233
Number of Diseased Vessels	2.47 (0.71) (n=1182)	2.81 (0.70) (n=43)	0.003	1225
1	11.4% (136/1192)	4.6% (2/44)	0.22	1236
2	30.7% (366/1192)	20.5% (9/44)	0.20	1236
3	55.8% (665/1192)	61.4% (27/44)	0.56	1236
>3	1.27% (15/1182)	11.6% (5/43)	<0.001	1225
SYNTAX score	27.9 ± 12.4 (n=817)	31.8 ± 14.3 (n=32)	0.084	849
Ischemia Jeopardy Score	8.9 ± 2.1 (n=940)	8.9 ± 2.4 (n=34)	0.99	974

Data presented as n (%) or mean ± standard deviation, where applicable. *eGFR was calculated using 2021 CKD-EPI Creatinine Equation. †Includes severe aortic stenosis/regurgitation and severe mitral stenosis/regurgitation. CRT denotes cardiac resynchronization therapy; eGFR, estimated glomerular filtration

rate; ICD, implantable cardioverter-defibrillator; LM, left main; PCI, percutaneous coronary intervention; PM, pacemaker; SYNTAX, the Synergy between PCI with Taxus and Cardiac Surgery.

Supplemental Table S9. Multivariable models for patients with hypotensive episodes.

**N=1,236**	**HR (95% CI)**	**p Value**
Medical history		
Chronic kidney disease	1.92 (1.02, 3.59)	0.04
Admission Characteristics		
Acute myocardial infarction on admission	1.95 (1.04, 3.65)	0.03
Angiography Characteristics		
Graft disease	4.06 (1.44, 9.89)	0.003
Number of Diseased Vessels	2.12 (1.26, 3.80)	0.007
Impella Characteristics		
Impella access: Subclavian/Transaxillary/Transcaval	2.76 (1.00, 6.51)	0.03

Supplemental Figure S1. Proportions of missing data per variables

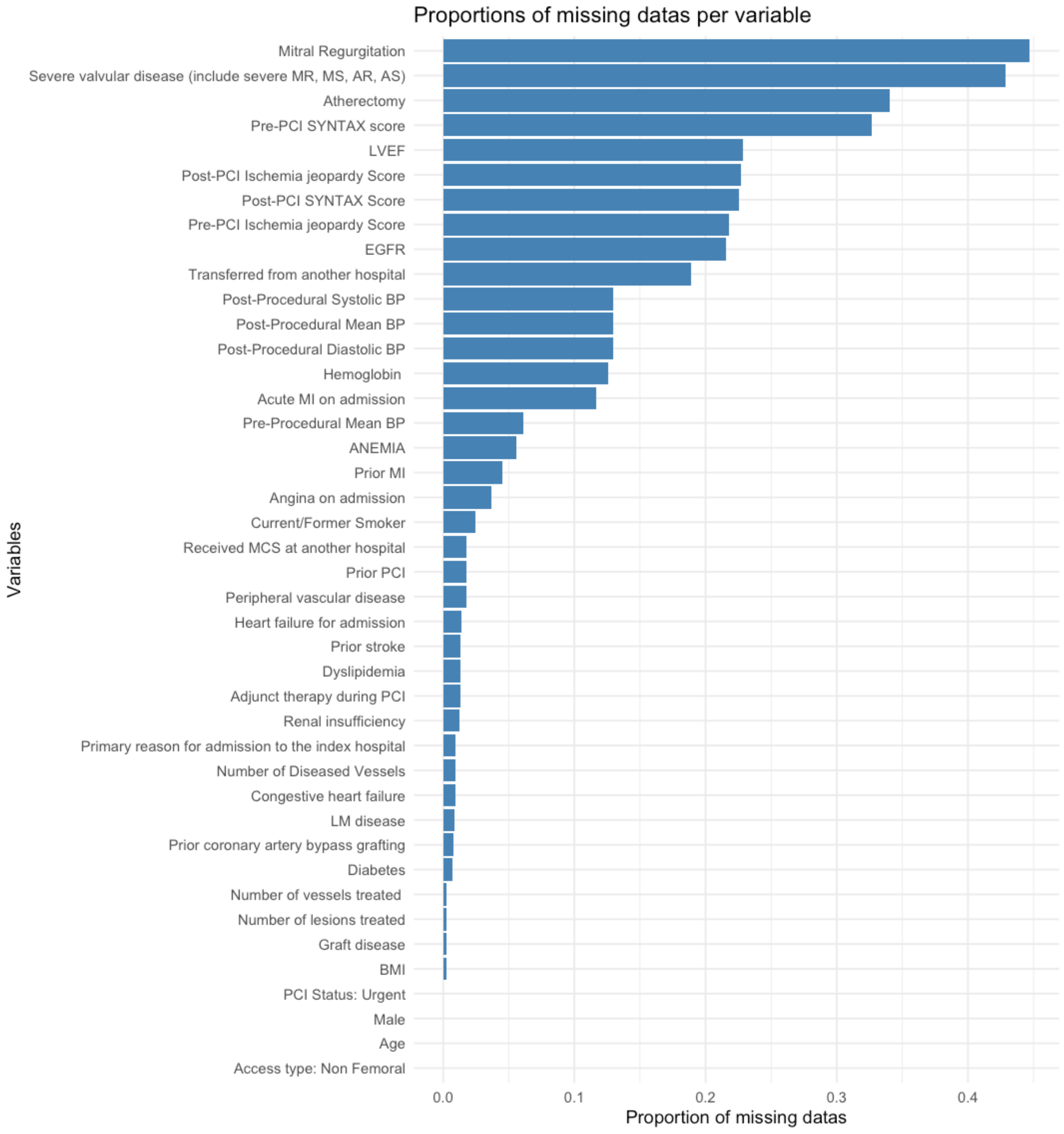

Supplemental Figure S2. Study flowchart.
MBP denotes mean blood pressure.

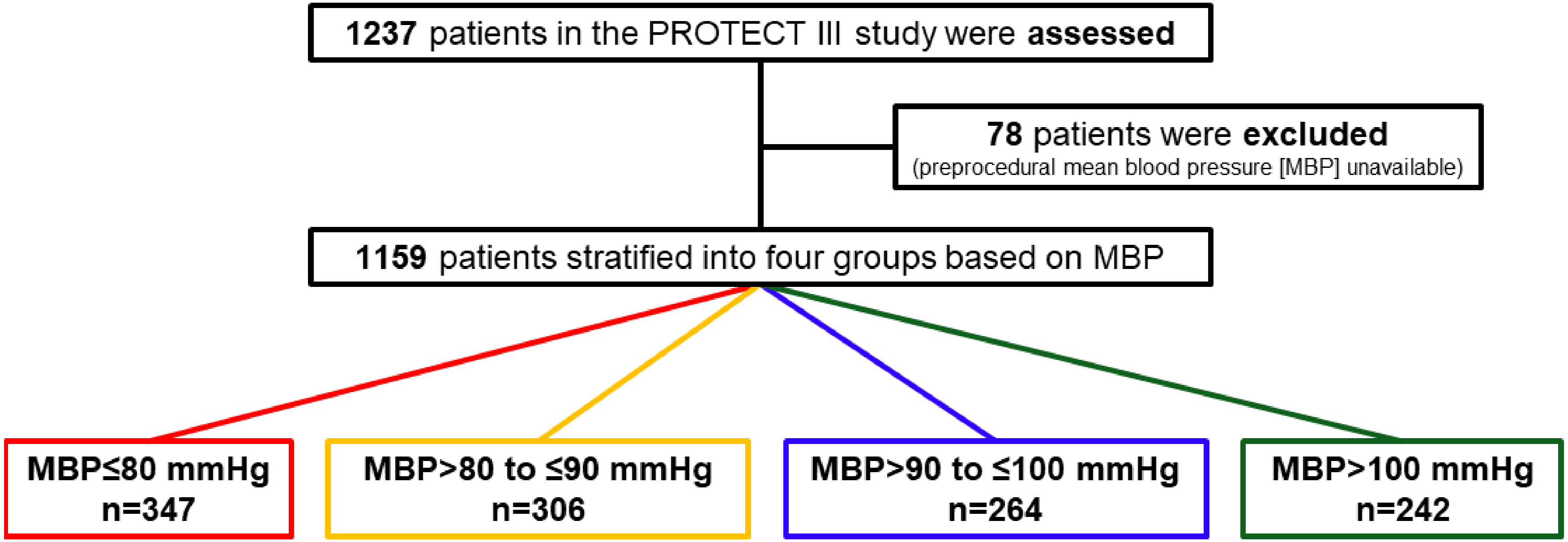

Supplemental Figure S3. Kaplan Meier Curves for nonfatal components of 90-day MACCE

A. Myocardial Infarction

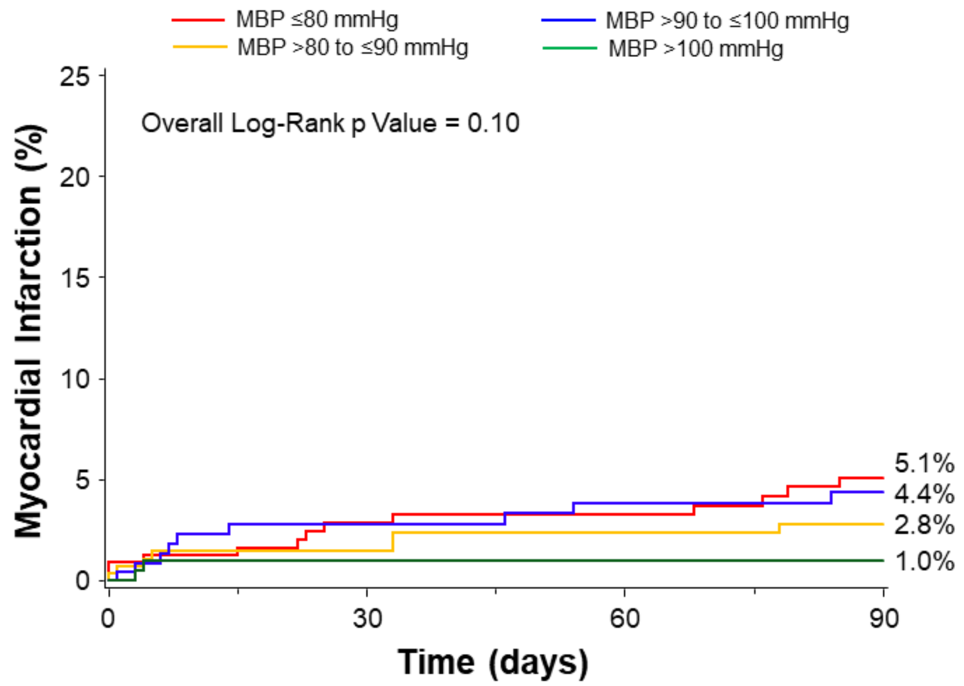

Number at risk:

MBP ≤80 mmHg	347	233	214	203
MBP >80 to ≤90 mmHg	306	227	215	204
MBP >90 to ≤100 mmHg	264	189	181	178
MBP >100 mmHg	242	182	175	172

B. Stroke/TIA

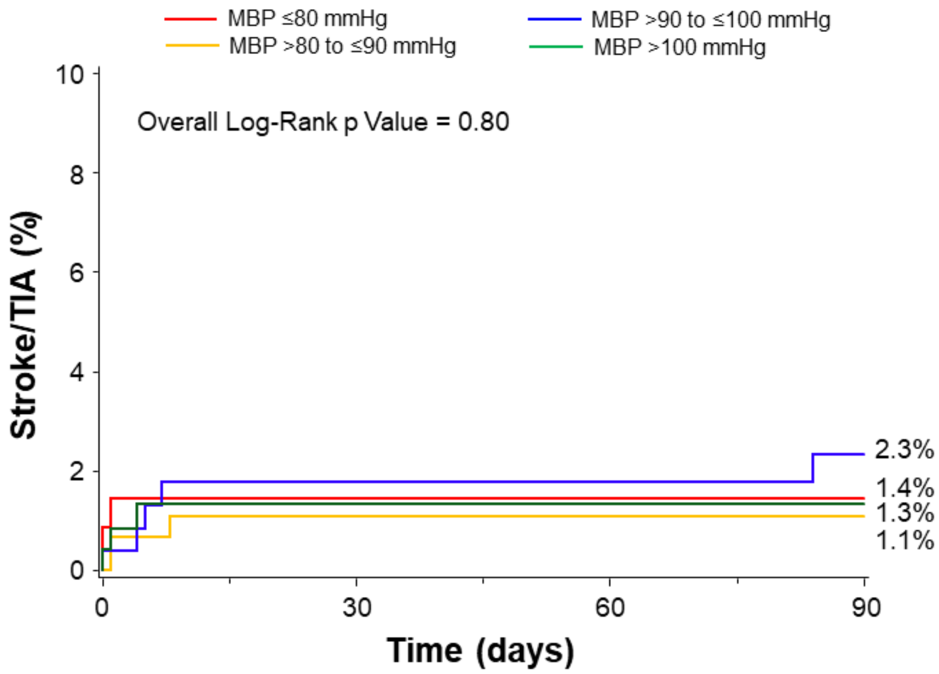

Number at risk:

MBP ≤80 mmHg	347	236	217	206
MBP >80 to ≤90 mmHg	306	227	216	206
MBP >90 to ≤100 mmHg	264	189	181	177
MBP >100 mmHg	242	181	174	171

C. Repeat Revascularization

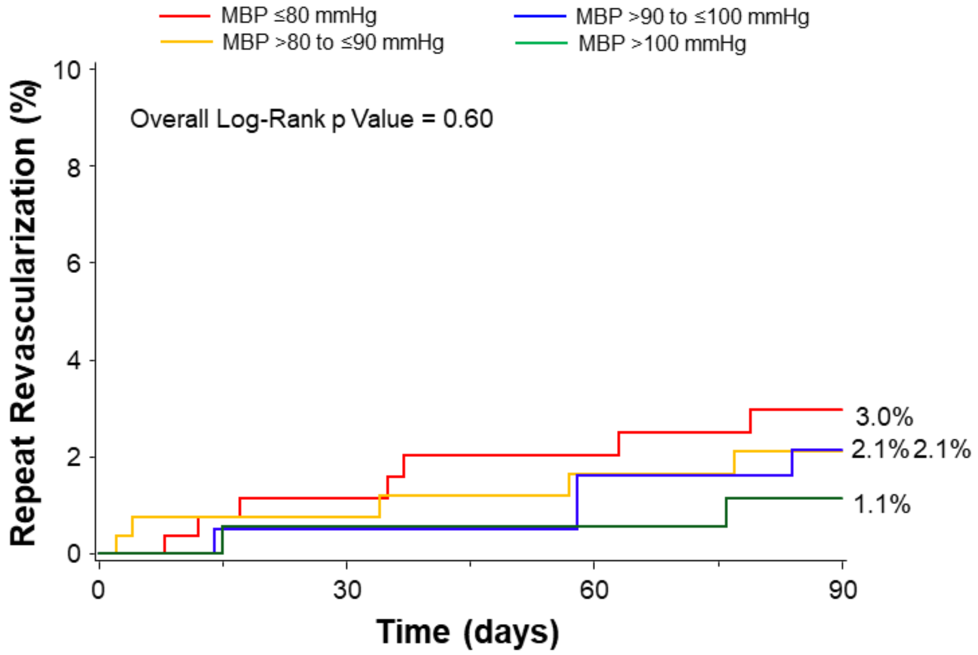

Number at risk:

MBP ≤80 mmHg	347	233	212	200
MBP >80 to ≤90 mmHg	306	228	215	204
MBP >90 to ≤100 mmHg	264	191	181	177
MBP >100 mmHg	242	181	174	170
